# Large right middle cerebral artery stroke with hemorrhagic transformation

**DOI:** 10.1186/s12245-024-00739-6

**Published:** 2024-10-04

**Authors:** Jack Niles, Garv Bhasin, Latha Ganti

**Affiliations:** 1 Trinity Preparatory School, Winter Park, FL USA; 2 Brown University, Providence, Rhode Island, USA; 3 Orlando College of Osteopathic Medicine, Winter Garden, FL 34787 USA; 4 Warren Alpert Medical School of Brown University, Providence, RI 02903 USA

**Keywords:** Right middle cerebral artery stroke, Ischemic stroke, Stroke thrombolysis, Hemorrhagic transformation

## Abstract

The authors present a case of an acute right middle cerebral artery infarct in a 65-year-old male with a history of diabetes, hypertension, and cardiovascular disease. The timeline of treatment and the evolution of the stroke is described. This case highlights the significant burden of right-sided cerebral artery stroke, even when intervention is swift.

## Introduction

A stroke occurs when the blood supply is cut off from a certain part of the brain depriving it of oxygen and resulting in the death of brain cells. There are two main types of strokes, ischemic and hemorrhagic. Ischemic strokes are a result of a blood clot occluding an artery, preventing blood from perfusing the brain territory supplied by that artery, while hemorrhagic strokes are the result of bleeding in a given territory or distribution. Middle cerebral artery (MCA) infarcts are often devastating, with many patients rarely regaining full motor function. MCA infarcts are usually caused by a thrombus or embolus occluding the middle cerebral artery [1].

Stroke is the fifth leading cause of death in the United States with about 160,000 deaths in 2020 [2]. Stroke occurrences and fatalities are becoming less common in high-income countries while remaining stagnant in middle or low-income countries. This is likely related to improvements in stroke prevention in high-income countries. The burden of stroke increases with age due to an increase in hypertension and dyslipidemia prevalence. The incidence of stroke is 2.6% in adults over 20 years old in the U.S. Ischemic strokes account for about 85% of all strokes and about half of those are located in the MCA. Young and middle-aged men have a higher prevalence of stroke than women, while overall, women have a higher prevalence of stroke throughout their whole lives; the risk of stroke in women is 20–21% while it is 14–17% in men [3]. The main risk factors for stroke are heart disease, history of stroke, past stroke, coronary artery disease, hypertension, and hypothyroidism [4].

Neurological symptoms such as unilateral weakness or numbness, facial droop, and speech impairment including dysarthria and aphasia are characteristic of MCA strokes [5]. The differential diagnosis for stroke includes several stroke mimics such as seizures, migraines, syncope, sepsis, and functional etiologies.

## Case presentation

**Fig. 1:**
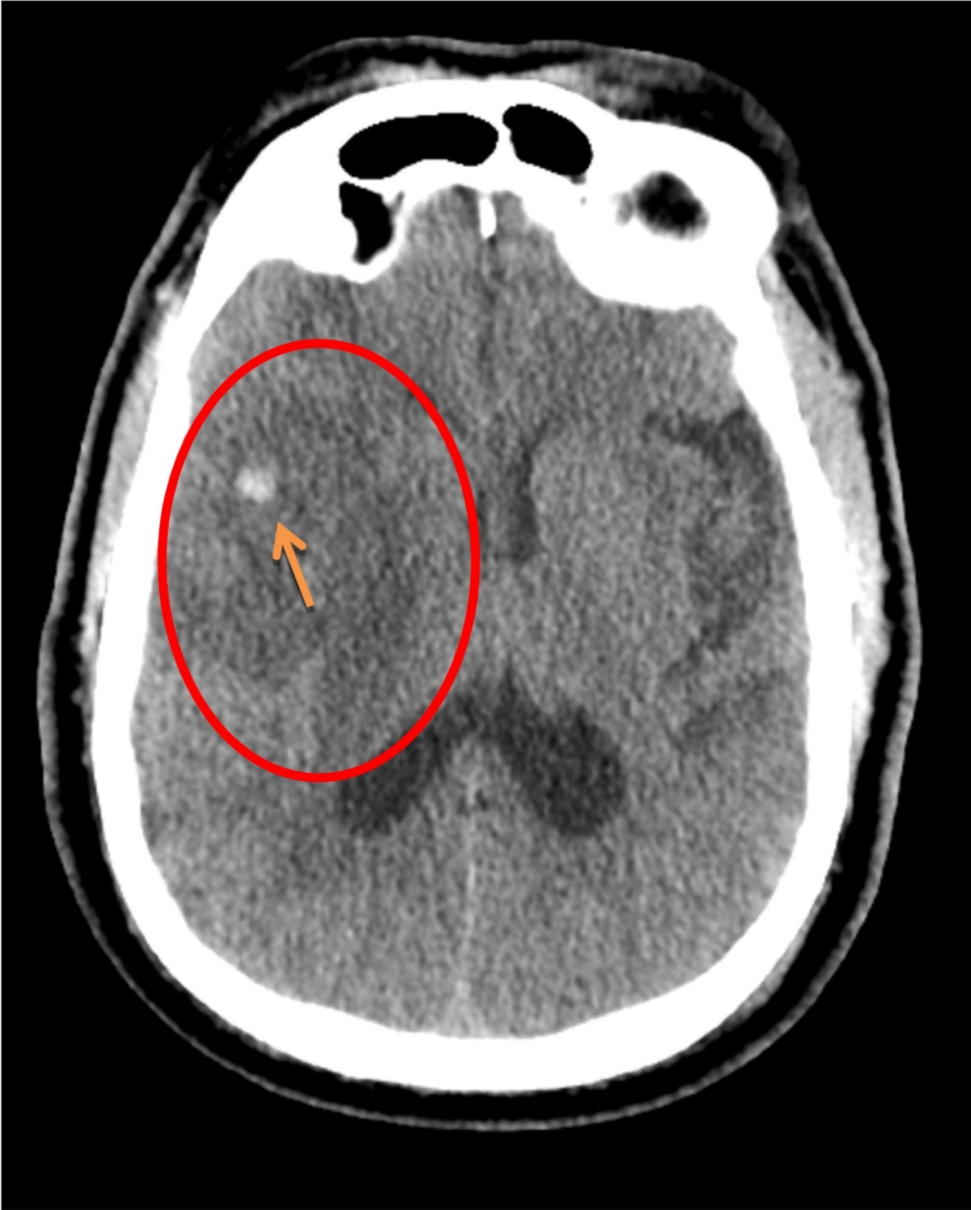
Noncontrast brain CT demonstrating large hypodensity corresponding to right MCA infarct (red circle). Within the area of infarct is a small hemorrhagic focus (orange arrow)

The patient is a 65-year-old, male who was brought in by Emergency Medical Services (EMS). His wife reported that he was in his usual state of health eating breakfast that morning, when he began experiencing sudden left-sided weakness, expressive aphasia, and a fairly sudden decreased level of consciousness. His past medical history was significant for hypertension, diabetes, and prior myocardial infarction. Upon arrival at the emergency department (ED), his temperature was 98.50^0^C, blood pressure 160/100 mmHg, heart rate 82 beats/minute, and respiratory rate 20 breaths/minute. His pulse oximetry was 93% on room air, and his fingerstick glucose measurement was 180 mg/dL. His National Institute of Health Stroke Scale (NIHSS) upon arrival was 15. He reported nausea, and vertigo, but did not vomit. Head CT was negative for hemorrhage while CT Angiogram of the head and neck showed a proximal right MCA cutoff and right MCA large vessel occlusion. Exclusion criteria were reviewed with the patient’s wife, and the patient was thrombolysed with tenecteplase and taken for mechanical thrombectomy. The following day, his NIH scale worsened to 25. Repeat head CT re-demonstrated the large MCA infarct, with a small focus of hemorrhage within the infarct, indicative of an evolving infarct [Fig. 1]. His NIHSS did not improve much over the next few days, but he was stable enough to be discharged to a skilled nursing facility on hospital day 7.

## Discussion

**Fig. 2:**
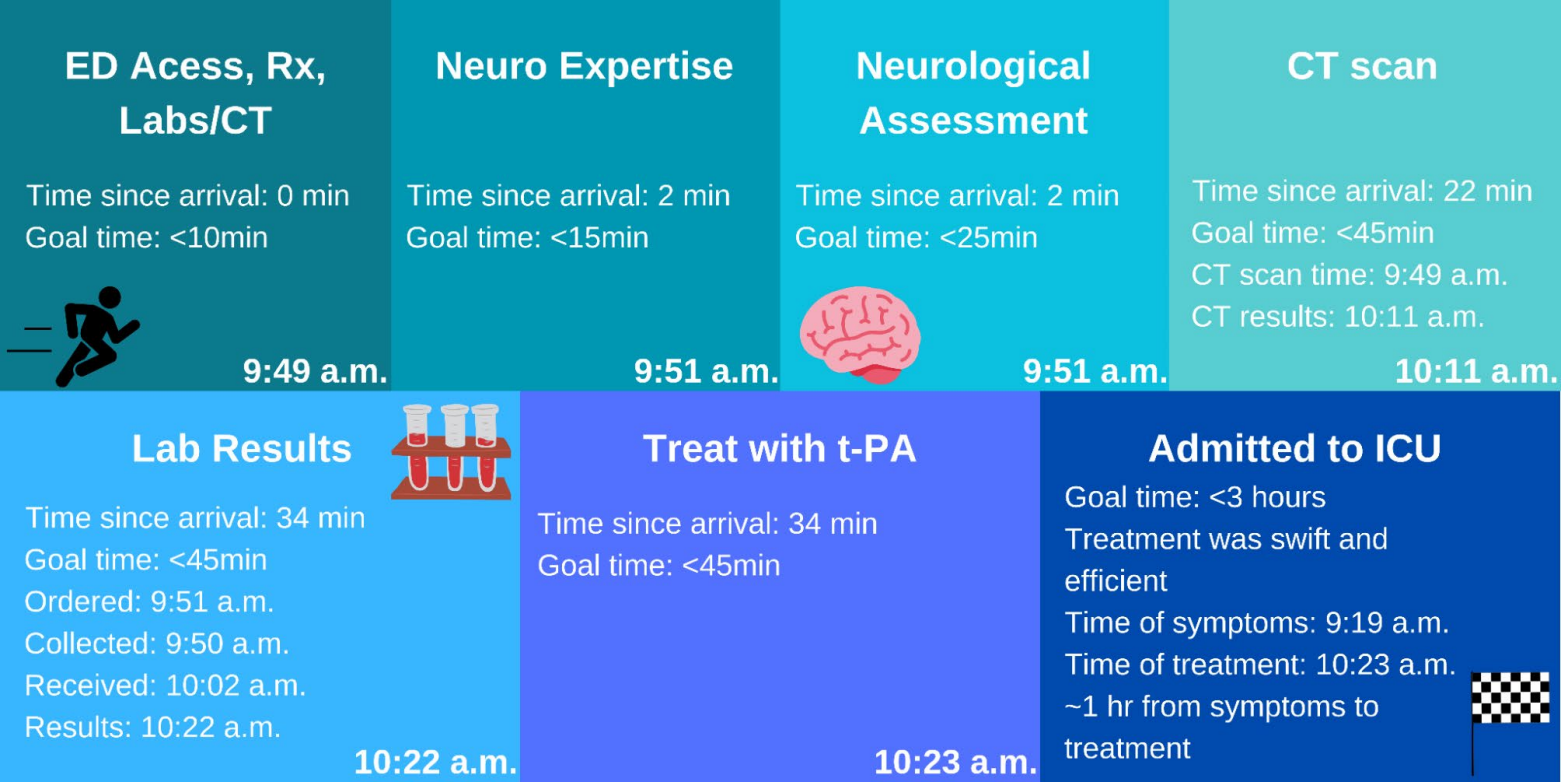
Infographic depicting the patient’s timeline designed by Jack Niles n Canva.com.

Acute stroke results in the loss of 1.2 billion neurons every second [6], thus the adage “time is brain!” In this case, the timeline from symptom onset to administration of tenecteplase was a swift 34 min, with all interim metrics met. [Figure 2].

The patient was also fortunate that he was already at a comprehensive stroke center which meant he could undergo prompt thrombectomy. A study of data from a hub-and-spoke academic telestroke network found that potential thrombectomy candidates with longer transfer times had lower odds of undergoing endovascular treatment. This probability further decreased by 1% for each additional minute of transfer time over 60 min [7].

The bleeding risk associated with thrombolytic therapy for acute ischemic stroke is typicall quoted as 6% [8]; however, certain comorbidities increase that risk. A systematic review of 55 studies that measured 43 baseline variables in 65 264 acute ischemic stroke patients found that the risk of hemorrhage after tPA increased with older age (odds ratio, 1.03 per year; 95% confidence interval, 1.01–1.04), higher stroke severity (odds ratio, 1.08 per National. Institutes of Health Stroke Scale point; 95% confidence interval, 1.06–1.11), and higher glucose (odds ratio, 1.10 per mmol/L; 95% confidence interval, 1.05–1.14). Our patient was aged 65 years, with diabetes—so perhaps at somewhat increased risk [9].

Following the thrombectomy, our patient was transferred to the ICU where secondary stroke prevention measures were implemented. Our patient already had three major comorbidities associated with stroke, including hypertension, diabetes, and cardiovascular disease. Medications for these conditions were reviewed and optimized.

The patient’s acute presentation with an NIHSS score of 15, a 10-point worsening within 24 h, CTA confirming a proximal MCA occlusion, and hemorrhagic transformation is consistent with a malignant middle cerebral artery (MMCA) infarction [10]. A systematic review of 2673 patients across 73 studies found that while mortality rates between right and left MMCAs were similar, the morbidity associated with right sided MMCAs was worse [11].The authors also reported that MMCAs were seen more frequently on the right, as was the case with out patient.

While left hemispheric strokes are more frequent than right sided ones [12] right sided MCA strokes, can have an extreme impact on one’s ability to process emotion as well as facial expressions. Heilman et al. showed that almost all patients who had large strokes on the right side of their brain couldn’t identify facial expressions or process their own emotions [13]. This can impact both inpatient and home care. Specifically, right-side MCAs will affect the limbic and paralimbic systems in the brain, greatly diminishing the amount of happiness a person is able to feel [14]. Interestingly, a study of 6 MCA stroke patients and 12 age and sex-matched controls revealed that MCA stroke patients experienced diminished response to positive stimuli while showing no change in response to negative stimuli [15]. The patient would also present this symptom when in inpatient physical therapy. This makes for caregiving challenges for these patients who already experience significant limitations in their activities of daily living.

### Limitations and future directions

One of the limitations of our case is the lack of availability to the echocardiogram report. MCA strokes are generally categorized as large artery strokes per the TOAST classification [16], rather than a cardioembolic source, but an echocardiogram is still part of the stroke workup and may have revealed other pathology terms of future directions, further prospective studies on mitigating the morbidity associated with MMCA strokes could shed light on decreasing the significant morbidity associated with this type of cerebral infarction.

## Conclusion

This case represents a malignant middle cerebral artery infarct that was treated promptly with the best available therapy and unfortunately still resulted in devastating deficits secondary to a large area of infarct. Malignant middle cerebral artery strokes represent a significant burden for stroke survivors.

## Data Availability

No datasets were generated or analysed during the current study.
